# Job morale: a scoping review of how the concept developed and is used in healthcare research

**DOI:** 10.1186/s12889-020-09256-6

**Published:** 2020-07-25

**Authors:** Alina Sabitova, Lauren M. Hickling, Stefan Priebe

**Affiliations:** 1 Unit for Social and Community Psychiatry, World Health Organisation Collaborating Centre for Mental Health Development, Queen Mary University of London, London, E13 8SP UK; 2 East London NHS Foundation Trust, Newham Centre for Mental Health, London, UK

**Keywords:** Job morale, Healthcare, Assessment, Model, Framework, Concept, Morale, Development

## Abstract

**Background:**

The job morale of healthcare staff is widely seen as an important factor for the quality of care. Yet, there are different understandings of what constitutes job morale, which hinders systematic research and comparisons. We therefore conducted a scoping review of how the concept of job morale has developed over time and how it is used in healthcare research.

**Methods:**

A scoping review was conducted to identify relevant literature. Data were gathered on study design and context, objectives, definitions of morale, outcome measures and key findings. Data was synthesised using a descriptive analytical framework.

**Results:**

Ninety-three unique studies met eligibility criteria for the present review. The literature outlines four main periods of the evolution of the concept of job morale: The First World War and the interwar years; Second World War; Aftermath of the Second World War; and Contemporary period. The concept of job morale originated in a military context and was later applied to and specified in the healthcare literature. The concept has been applied to individuals and groups. The understandings used in healthcare vary, but overlap. Methods for assessing job morale in healthcare include quantitative scales, indirect measurements of consequences and predictors of morale, and qualitative approaches. Existing studies have mainly focused on the job morale of general practitioners, nurses and mental health professionals in high-income countries.

**Conclusions:**

Although the understandings of job morale in healthcare are heterogeneous and inconsistent, the concept appears to have been useful over longer periods of time and in different contexts. Which precise understanding of job morale is useful, depends on the given research purpose, and studies should make explicit which exact understanding they apply. Systematic research on job morale is required to facilitate measures to improve and maintain high levels of morale across different professional groups, including professionals in low- and middle-income countries.

## Background

‘Job morale’ is a term that has been used both in healthcare and in wider contexts. This interest is caused by a widespread assumption that job morale can significantly influence performance [1]. Evidence suggests that healthcare staff with positive job morale are more likely to provide higher quality care to patients [2, 3], and it is suggested that improving job morale could improve job performance address inadequate job performance in areas with fewer/inadequate resources [4]. Furthermore, positive job morale is associated with greater retention and higher recruitment in healthcare staff [5].

Despite its importance, applied understandings of job morale in the healthcare context vary from study to study, suggesting that it is “not a well-defined or precisely measured concept” [6]. A number of authors have tried to explore job morale as a single entity, but ended up assessing its predictors, consequences or explanatory variables, such as job motivation, job satisfaction, burnout, organizational commitment, work engagement and well-being [5–9]. The terminology is inconsistent, with terms such as ‘morale’ and ‘job morale’ used interchangeably, although just ‘morale’ is a wider term and not necessarily job-related. Some terminological confusion seems to be linked to the history of the term. ‘Job morale’ stems from the general ‘morale’ concept, which has changed over time. In some instances, job morale is synonymously used with other job-related concepts, such as job satisfaction, well-being or job motivation. Historically, this equation was supported by the fact that readers were referred to job satisfaction when searching for morale in the Index of Psychological Abstracts, (a periodical of indexes and abstracts in psychology, the print version of the PsychInfo database, which was ceased in 2006), between 1970 and 1972 [10]. In 1978, the concept of morale was reintroduced to the Index of Psychological Abstracts after being delisted in 1973 [10] as equal to job satisfaction, causing a resurgence in the topic of morale in both job and non-job-related contexts. A recent systematic review and meta-analysis into the job morale of physicians and dentists in low- and middle-income countries (LMIC) [11] indicated a substantial methodological heterogeneity across studies measuring job morale. This heterogeneity compromises attempts to justify its importance and to evaluate the impact of job morale on performance and make comparisons across different contexts and countries. There is very little unifying research on what job morale is and how it should be assessed, particularly in the field of healthcare [12].

Considering the heterogeneity of the scientific literature on job morale in the healthcare context, we aimed to conduct a scoping review of how the concept of job morale has developed over time and how it is used in healthcare research. In particular, we wanted to review which theoretical frameworks have been applied to job morale in healthcare, how job morale has been assessed, and the professional groups in which job morale has been studied.

## Methods

### Design

A scoping review was considered the most appropriate method to capture the concept of job morale, address the broad research question and inform future research [13]. This review is reported according to the Preferred Reporting for Systematic Reviews and Meta-Analysis Extension for Scoping Reviews (PRISMA-ScR) guidelines.

### Search strategy

A search with no date limitation was conducted for the term ‘morale’ within titles and abstracts in PubMed, and titles in Scopus and EMBASE. In order to capture the evolution of job morale across contexts, the search was deliberately broad. Unpublished literature was searched using Google Scholar and OpenGrey, in an attempt to reduce publication bias, and backward and forward citation tracking was adopted. Furthermore, hand searches were performed in two special issues that were dedicated to the topic of morale, in the American Journal of Sociology (Volume 47, Number 3, November 1941) and Journal of Educational Sociology (Volume 15, Number 4, December 1941).

### Study selection

#### Two sets of eligibility criteria were applied

In order to address the development of the concept, studies were included if they met one of the following criteria: [1] review studies explicitly reporting a meaning or framing of the concept of morale prior to 1978; [2] quantitative and/or qualitative studies assessing morale prior to 1978; [3] review studies explicitly reporting a meaning or framing of job morale after 1978; or [4] quantitative and/or qualitative studies assessing job morale after 1978. Studies were excluded if: [1] they were published in a language other than English; [2] were non-research based articles (such as commentaries, conference abstracts, editorials or book chapters); [3] the abstract or full text was not available.

To best meet the aims of the review we adopted two sets of inclusion criteria depending on the year of publication, informed by the history of the concept. To capture the emergence and historical evolution of the concept of morale, we included papers considering morale across contexts and not specifically focusing on “job morale”. The year 1978 was chosen as the threshold for the beginning of the contemporary period of morale research. As reported in the introduction, the concept of morale was reintroduced to the Index of Psychological Abstracts, causing the resurgence of interest in the topic in both job-related and non-job-related contexts. Considering the primary interest of the current review in job morale, the inclusion for the contemporary literature (i.e. studies published after 1978) was narrowed to studies exploring job morale.

To meet the second aim of the review, assessing how the concept of job morale has been used in healthcare research, the inclusion criteria were narrowed to [1] studies explicitly reporting a meaning or framing of job morale in the healthcare context after 1978; or [2] quantitative and/or qualitative studies assessing the job morale of any healthcare staff after 1978. Studies were excluded if they were published prior to 1978 or were conducted in contexts other than healthcare.

### Review strategy

EndNote X8 (Clarivate Analytics) was used to screen the titles and abstracts of identified studies. Duplicate and irrelevant studies were excluded. Full-texts were inspected against the inclusion criteria by the primary reviewer (AS), and a random subset of 20% of these were independently screened by a second reviewer (LMH) at each stage. Discrepancies were solved by consulting a third reviewer (SP). Data gathered included the study design, objectives, context, definition of morale (if reported), outcome measures and main findings.

### Data charting process

A draft of the data charting form was created and piloted for fifteen of the included studies. Minor changes were made accordingly. The following categories were included in the final form and applied for all included studies: author(s), year of publication, country, study design, study context, main objectives, the definition of morale (if any), study population, methodology, outcome measures and main findings.

### Data synthesis

A descriptive analytical method was used, in line with Arksey and O’Malley’s framework [13]. The identified literature was mapped according to the main development periods that the concept of job morale has undergone. Then the literature on job morale in the healthcare context was organised, highlighting the models and theoretical frameworks of job morale, the assessment methods and the professional groups studied.

## Results

**Fig. 1:**
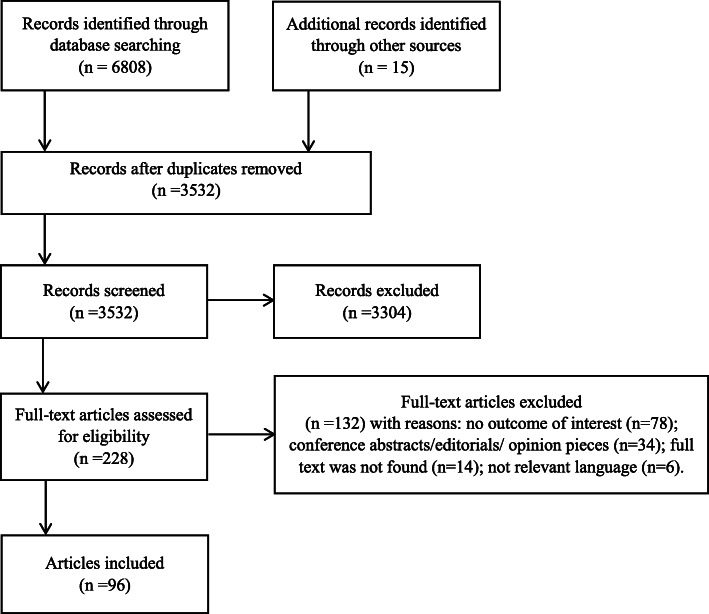
PRISMA-ScR flow diagram

A total of 6808 articles were identified through database searches, and 15 through other sources. Overall, 3304 articles were excluded as duplicates, and 3304 additional articles were excluded for not meeting the inclusion criteria. Two hundred and twenty-eight full texts were examined, of which 96 were included, representing 93 unique studies (Fig. 1).

### Study characteristics

The included studies were published between 1941 and 2019, and most studies were conducted in the United States of America (USA, *n* = 48) and the United Kingdom (UK, *n* = 24). With regards to study design, the most prevalent type was quantitative (*n* = 37), followed by reviews (*n* = 36). Further study characteristics can be seen in Additional File 1.

The results of the current review will be presented in two parts. In the first part, the historical evolution of the concept of job morale will be introduced. In the second part, contemporary job morale research in the healthcare context will be presented.

#### Historical development of the concept of job morale

The literature outlines four main periods of the evolution of the concept of job morale: [1] The First World War (WWI) (from 1914 to 1918) and the interwar years (from 1918 to 1939); [2] Second World War (WWII) (from 1939 to 1945); [3] Aftermath of WWII (from 1945 to 1980s); [4] Contemporary period (since 1980s). WWI and the inter-war years marked the period where early research in morale began, as the shift from a limited number of professional soldiers to an expansion of armies recruited from civilians was required [14]. Therefore, creating “the necessary offensive spirit” [9] among civilian armies was vital, to keep them combative, aggressive and vigilant. Attempts to improve morale were taken using training, referred to as the “psychological battlefield”, monitoring the sickness rates of soldiers and observing letters home [15]. During the interwar years, interest in military morale lessened, and civilian morale became more of a concern [16]. Baker [17] suggested that civilian morale was “a group phenomenon”, which “emphasizes the spirit of the agreement and of co-operative action” towards goals.

The outbreak of WWII led to a resurgence of interest in military morale, but was also explored from a civilian perspective [18–22]. With peacetime after WWII came a shift from the civilian/militaristic perspective of morale to the view of employees. Employee morale was examined across multiple industries [23–25]. It was measured using questionnaires and surveys, the validity and generalisability of which however was rarely assessed [26, 27].

Since in the 1980s, a clearer terminology emerged, as Payne et al. specified a difference between organisational and job morale. Job morale was proposed as the satisfaction of a group with the job itself, whereas organisational morale was the satisfaction of a group with the organisation [28]. McKnight et al. described job morale as “the degree to which an employee feels good about his or her work environment” [29]. Job morale was associated with other factors, such as performance and productivity [30, 31], work effort [30], intention to leave [6] and customer satisfaction [32]. Weakliem and Frankel [30] defined job morale as “a general term for positive feelings about the job”. Motivation and job satisfaction were parts of the different definitions of job morale in healthcare, where job morale was defined as “a general term encompassing the main aspects of work-related well-being and satisfaction and engagement with work”.

#### Job morale in healthcare research

##### Theoretical frameworks and models

Bedian and Armenakis [33] proposed one of the first models, proposing that conflict and ambiguity in job roles was associated with decreased job satisfaction, which was associated with a propensity to leave the organisation. This model was utilised in a cross-sectional study on nurses and was used to explain changes in morale levels of healthcare staff during the integration of mental and social care services in England [8].

Alternatively, Warr [34, 35] developed a framework around affective well-being, which outlines three dimensions of well-being: pleasure to displeasure, contentment to anxiety, and enthusiasm to depression [35]. When viewed from a job context the positive ends of the dimensions indicate satisfaction with the employees’ job, whereas the negative ends indicate burnout [35]. This framework was used in a mixed-methods study conducted by Johnson et al. It aimed to explore the job morale of mental health staff in an inpatient setting [9], and concluded that employees need to be pleased, enthusiastic and comfortable with their job in order to have positive job morale, as opposed to displeasure, anxiety and depression [9].

A commonly applied theory is the Job Demand-Control (JD-C) Model [36], which considers the decision making capability and demand of employees to be determinants of job-related strain. More specifically, job morale depends on the employees’ autonomy to make decisions in the workplace and the breadth of skills they can utilise, as well as psychological stressors in the workplace. The model hypothesises that high levels of demand and decision capability leads to high job motivation, satisfaction and thus positive job morale [37]. This model was used to inform the analysis of qualitative findings exploring the impact of institutional and social changes on GPs’ morale in England [38]; to define job characteristics related to morale in a national investigation of inpatient mental health staff morale in England [9] and to develop a model of work-related morale for nursing assistants in Taiwan [39].

Siegrist’s Effort-Reward Imbalance (ER-I) Model [40] considers the effects of effort and reward on job-strain and job morale. Efforts relate to job demands and obligations, and the inherent motivation to meet them. Rewards include financial profit, benefits to esteem, job security and career opportunities [40]. The ER-I model hypothesises that an imbalance between reward and effort causes job strain, decreases employee well-being and provokes negative job morale [40, 41]. This suggests that a balance between effort and reward is needed for positive employee well-being and job morale. The Job Demands-Resources (JD-R) Model [42] was proposed to encompass factors associated with job satisfaction [43], work related well-being [44, 45] and burnout [46]. It incorporates the physical, social, psychological and organisational aspects of a job, and resources that can be used to address or reduce job demands. The model suggests that combining high demands and limited resources leads to job strain [42].

### Assessment

Single-item scales have been widely used for asking respondents to rate their job morale. One of the most extensive staff surveys in the world was conducted by the National Health Services in England [47]. Staff were asked to rate their job morale on a scale from 0 to 10. Single item scales have also been adopted by several researchers to define job morale among medical students [48], nurses [48, 49], working mothers in obstetrics and gynaecology [50] and GPs [51–53]. In this case, the definition of job morale can be interpreted by the respondent, who may have varying understandings of the term. Multiple item scales have also been employed, such as the Hospitalist Morale Index (HMI) [54] and Morale Assessment in the General Practice Index (MAGPI) [55]. They were developed and refined using exploratory and confirmatory factor analyses [55] and principle components analysis [54] respectively. Despite this, the content validity of these scales is still problematic, which could be one of the reasons for why these measures have not been used more widely.

As well as using direct measures, job morale can be assessed by inferring morale from its consequences and predictors. For example, absenteeism, staff sickness, turnover and patient satisfaction are often taken to be consequences of low job morale [1, 56, 57]. Such variables are commonly accessible to employers, but they may also reflect changes in working conditions and industry fluctuations and not just job morale. Indicators that have been considered to be predictors of job morale varied across studies and often overlapped with those variables considered to be consequences, (for example, staff turnover) thus questioning the content validity of the quantitative scales.

In the light of the limitations of quantitative measures of job morale, several authors have applied qualitative methods to explore job morale [38, 58–62]. Such methods, mostly in the form of interviews and focus groups, have been used flexibly and contributed to theory building, adopting an inductive approach without labelling predefined variables as job morale.

### Research focus

Research on job morale in healthcare has focused on healthcare students, GPs, nurses and mental health professionals. Chen [59] described job morale in nursing students to be a “mental and emotional condition of a person or a group with regard to its function, which is exhibited by a disposition marked by confidence, cheerfulness, discipline, and willingness that drives the individual desire to succeed”. Job morale in students has been assessed in several studies without attempting to define the concept [48, 63, 64], and mostly adopted qualitative methods [59, 65]. However, the morale of students is also likely to be influenced by their satisfaction with the educational component of being a student and cannot be considered as entirely job-related.

The job morale of GPs has been explored in a number of UK based studies [1, 38, 51, 52, 61, 62, 66, 67], most of which did not use a definition of the concept [1, 38, 51, 61, 62, 66, 67]. Grieve commented that one of the main job morale indicators is well-being [1], and Gilliland et al. defined job morale as being “feelings of confidence in one’s situation with a positive hope for the future” [52]. A single-item Likert scale was adopted by studies using a cross-sectional design [51, 52]. Low levels of job morale were reported by the majority of respondents [51, 52], and several qualitative studies identified underlying causes of positive and negative job morale, including workload, changes to the work environment, decreased autonomy, increased work demand and fragmented teams [38, 61].

Amongst nurses, morale was described to be “the degree to which an employee exhibits a positive or motivated psychological state. It can manifest itself as pride in the organisation and its goals, faith in its leadership, and a sense of shared purpose with, and loyalty to, others in the organisation” [68]. In a review, Day et al. stated that “positive morale is seen as an attitude of confidence in the mind of the individual where they identify with a group, accept group goals and work towards achieving them collectively” [12]. The authors went on to analyse personal and organisational job morale as separate entities, suggesting that there is a difference between group and individual morale [69]. The studies assessing job morale in nursing have adopted both single and multiple-item Likert scales, and from these it has been suggested that job morale in this group is associated with work environment [49], turnover [70], conflict resolution styles [71] and financial compensation [57].

Job morale has also been examined across mental health professions [72]. It was suggested that the term job morale is used as an umbrella term for a number of job-related variables, such as job satisfaction, burnout, occupational stress, psychological well-being, absenteeism, recruitment and retention issue and the incidence of psychiatric disorders [73]. This idea of job morale as an umbrella term was considered in some European studies [5, 74, 75]. It was suggested that job satisfaction, burnout and team identity are key indicators of morale, and that different levels of job morale depended on the professional group studied and the geographical area of the study [5, 74, 75]. Likewise, Johnson et al. conducted a mixed-methods national investigation of job morale in inpatient mental health staff across 19 mental health Trusts in England [7, 9, 58, 76]. Job morale was measured using a cluster of indicators, such as job motivation, satisfaction, well-being, burnout and overall psychological health [9]. Using these indicators, the authors concluded that staff job morale was good, but acknowledged that there were significant differences across service types [76]. Factors impacting on job morale were also explored qualitatively with both patients [58] and staff [7], with both groups expressing similar views. Both outlined the importance of a supportive working environment, clear organisational measures and close-knit teams [7, 58].

## Discussion

### Summary of evidence

The reports from 93 studies suggest that the concept of job morale originated from the military and gradually underwent four main periods of development up to the present day; [1] WWI and the interwar years; [2] WWII; [3] Aftermath of WWII; and [4] Contemporary period. Although there is no universally accepted definition of job morale, several overlapping key elements appear across definitions, such as group cohesion and job satisfaction. In research in healthcare job morale is seen either as an umbrella term that encompasses job-related variables or a unifying variable.

Job morale in healthcare has been assessed using direct and indirect as well as quantitative and qualitative methods. Scales have questionable content validity and the generalisability of the results is problematic. Job morale research in healthcare focused on GPs, nurses and mental health professionals, and was mostly conducted in western countries such as the UK and USA.

### Strengths and limitations

One of the important limitations of this scoping review is that the search strategy limited publications to the English language, covering articles published on health or health-related themes, which means that relevant studies conducted in other fields and languages were almost certainly missed. Furthermore, the quality of the studies was not assessed, and the provided data may have been influenced by different sources of bias. Despite these limitations, the current scoping review is the first comprehensive review on the topic of job morale. It employed a clear, extensive search strategy, providing a base for anyone who conducts explorations into job morale in the future.

### Differences of concepts and implications for research

The literature has used job morale as an individual and a group concept. The majority of the studies suggested that job morale is shared by members of the group. However, group members may have differing feelings about job morale at any one time, and this may differ from individual to individual. Job morale is associated with personality traits such as altruism [39] or neuroticism [77] which emphasises the individual variation. The most prevailing view is that job morale of a group is in part influenced by individuals in that group [16], therefore some authors chose to aggregate individuals’ job morale levels to measure group job morale [48–50, 64, 70]. Other authors proposed that job morale is simultaneously both an individual and group concept [59, 65] which required complex multi-level analytic methods in order to understand the interaction between individual and group job morale. In a study by Day et al. [69], the author attempted to model the individual and organisational job morale of nurses separately (individual job morale was listed as an outcome variable of organisational job morale, alongside other job-related variables, and vice versa). Job-related variables associated with individual and organisational job morale differed, supporting the view that it is useful to explore job morale both at group and individual levels [69].

The review identified significant gaps in the literature, specifically with respect to the origin of the studies and the assessment methods used. All of the studies included in the review except one [78] were conducted in high-income countries (HIC), as defined by the World Bank criteria [79]. This was expected considering that HICs commonly have more research and job resources than LMICs. According to the JD-R model, having limited job resources can play an important role in diminishing job morale, and future research should include LMICs to explore this further. With respect to professional groups, very limited research has been conducted on the morale of a wide range of relevant professional groups such as specialists other than GPs, nurses and mental health professionals. It was not clear from the existing literature why research was focused on these professional groups; therefore, we can only speculate about the reasons. It is important to note that the findings of the current review apply only to the studies that explicitly used the term job morale. As it was shown in a recent systematic review and meta-analysis [11], there are a number of studies on physicians working in LMICs assessing job-related variables encompassed by the term job morale, including job motivation, job satisfaction and burnout. Whilst some of these are quantitative in nature, there are also some qualitative studies in this area [80].

As job morale is often used as an umbrella term with varying specific understandings, future research on job morale in healthcare should specify how exactly they understand job morale, preferably providing a theoretical justification or their use of the term. For quantitative assessments, the dominating dimensions of high versus low and positive versus negative job morale appear useful and provide the options to use cut-off points for categorising scores into high, moderate or low morale. Such simplified categories may help using findings on job morale for policy makers, service development and on-going quality improvement processes.

## Conclusions

The concept of job morale has a long history and has been widely used. This may be due to its intuitive appeal, the applicability to different groups, its use as an umbrella term with varying components and its connotation that encompasses not only problems but also a positive meaning. Despite the heterogeneity in ways of understandings of the concept, it has guided meaningful research in healthcare.

Future research should specify which exact understanding of job morale is used, which may vary depending on the purpose of the given study. It should also advance the quality of the assessment methods, study all professional groups in healthcare and include professionals in LMICs. If this can be achieved, job morale is likely to continue to play a role in both future research and development of policies and practice in healthcare.

## Supplementary information

**Additional file 1.** Study Characteristics. A table contain study characteristics of all included studies.

## Data Availability

Not applicable.

## Abbreviations

ER-I: Effort-Reward Imbalance
GP: General Practitioner
HIC: High-Income Country
HMI: Hospitalist Morale Index
JD-C: Job Demand-Control
JD-R: Job Demand-Resources
LMIC: Low- and Middle-Income Country
MAGPI: Morale Assessment in the General Practice Index
PRISMA-ScR: Preferred Reporting Items for Systematic Reviews and Meta-Analysis Extension for Scoping Reviews
UK: United Kingdom
USA: United States of America
WWI: World War I
WWII: World War II

## References

[1] Grieve S (1997). Measuring morale - does practice area deprivation affect doctors’ well-being?. Br J Gen Pr..

[2] Department of Health (2009). NHS Health and Well-being: Final Report.

[3] Hall LH, Johnson J, Watt I, Tsipa A, O’Connor DB (2016). Healthcare staff wellbeing, burnout, and patient safety: a systematic review. PLoS One.

[4] Rowe AK, de Savigny D, Lanata CF, Victoria CG (2005). How can we achieve and maintain high-quality performance of health workers in low-resource settings?. Lancet..

[5] Reininghaus U, Priebe S (2007). Assessing morale in community mental health professionals: a pooled analysis of data from four European countries. Soc Psychiatry Psychiatr Epidemiol.

[6] Johnsrud LK, Heck RH, Rosser VJ (2000). Morale matters: midlevel administrators and their intent to leave. J Higher Educ.

[7] Totman J, Hundt GL, Wearn E, Paul M, Johnson S (2011). Factors affecting staff morale on inpatient mental health wards in England: a qualitative investigation. BMC Psychiatry..

[8] Gulliver P, Towell D, Peck E (2003). Staff morale in the merger of mental health and social care organizations in England. J Psychiatr Ment Health Nurs.

[9] Johnson S, Wood S, Paul M, Osborn DP, Wearn E, Lloyd-Evans B (2010). Inpatient mental health staff morale: a National Investigation. Final Report.

[10] Cooper CL, Jackson SE (1997). Creating tomorrow’s organizations: a handbook for future research in organizational behavior.

[11] Sabitova A, McGranahan R, Altamore F, Jovanovic N, Windle E, Priebe S (2020). Indicators associated with job morale among physicians and dentists in low-income and middle-income countries: a systematic review and meta-analysis. JAMA Netw Open.

[12] Day G, Minichiello V, Madison J (2006). Nursing morale: what does the literature reveal?. Aust Health Rev.

[13] Arksey H, O’Malley L (2005). Scoping studies: towards a methodological framework. Int J Soc Res Methodol.

[14] Gal R (1986). Unit morale: from a theoretical puzzle to an empirical illustration: an Israeli example. J Appl Soc Psychiatry.

[15] Jones E (2006). The psychology of killing: the combat experience of British soldiers during the first world war. J Contemp Hist.

[16] Child IL (1941). Morale: a bibliographical review. Psychol Bull.

[17] Baker HJ (1930). The maintenance of morale. Int J Ethics.

[18] Hocking WE (1941). The nature of morale. Am J Sociol.

[19] Ames ES (1941). Morale and religion. Am J Sociol.

[20] Hightower RL (1944). A sociological conception of morale. Soc Forces.

[21] Angell E (1942). Civilian morale: Democracy’s new line of Battle. J Educ Sociol.

[22] Griesser M (1942). Underlying factors in democratic morale. J Educ Sociol.

[23] Giese WJ, Ruter HW (1949). An objective analysis of morale. J Appl Psychol..

[24] Goode WJ, Fowler I (1949). Incentive factors in a low morale plant. Am Sociol Rev.

[25] Browne CG, Neitzel BJ (1952). Communication, supervision, and morale. J Appl Psychol.

[26] Webb WB, Hollander EP (1956). Comparison of three morale measures: a survey, pooled group judgments, and self-evaluations. J Appl Psychol..

[27] Campbell DT, Tyler BB (1957). The construct validity of work-group morale measures. J Appl Psychol..

[28] Payne RL, Fineman S, Wall TD (1976). Organizational climate and job satisfaction: a conceptual synthesis. Organ Behav Hum Perform.

[29] McKnight HD, Ahmad S, Schroeder RG (2001). When do feedback, incentive control, and autonomy improve morale? The importance of employee-management relationship closeness. J Manag Issues.

[30] Weakliem DL, Frenkel SJ (2006). Morale and workplace performance. Work Occup.

[31] Motowildo SJ, Borman WC (1978). Relationships between military morale, motivation, satisfaction, and unit effectiveness. J Appl Psychol..

[32] Abbott J (2003). Does employee satisfaction matter? A study to determine whether low employee morale affects customer satisfaction and profits in the business-to-business sector. J Commun Manag.

[33] Bedeian AG, Armenakis AA (1981). A path-analytic study of the consequences of role conflict and ambiguity. Acad Manag J.

[34] Warr P (2011). Work, happiness and unhappiness.

[35] Warr P (1990). The measurement of well-being and other aspects of mental health. J Occup Psychol.

[36] Karasek RA (1979). Job demands, job decision latitude, and mental strain: implications for job redesign. Adm Sci Q.

[37] de Jonge J, Kompier MAJ (1997). A critical examination of the demand-control-support model from a work psychological perspective. Int J Stress Manag.

[38] Napier J, Clinch M (2019). Job strain and retirement decisions in UK general practice. Occup Med.

[39] Wang YJ, Zhuang HL, Chiou JY, Wang CW, Wang CY, Liu LF (2018). Exploring factors influencing the work-related morale for certified nursing assistants in hospice care: a structural equation modeling study. PLoS One.

[40] Siegrist J (1996). Adverse health effects of high-effort/low-reward conditions. J Occup Health Psychol.

[41] van Vegchel N, de Jonge J, Bosma H, Schaufeli W (2005). Reviewing the effort-reward imbalance model: drawing up the balance of 45 empirical studies. Soc Sci Med.

[42] Bakker AB, Demerouti E (2007). The job demands-resources model: state of the art. J Manag Psychol.

[43] McVicar A (2016). Scoping the common antecedents of job stress and job satisfaction for nurses (2000–2013) using the job demands–resources model of stress. J Nurs Manag.

[44] Turnell A, Rasmussen V, Butow P, Juraskova I, Kirsten L, Wiener L (2016). An exploration of the prevalence and predictors of work-related well-being among psychosocial oncology professionals: an application of the job demands-resources model. Palliat Support Care.

[45] Spence Laschinger HK, Grau AL, Finegan J, Wilk P (2012). Predictors of new graduate nurses’ workplace well-being: testing the job demands-resources model. Heal Care Manag Rev.

[46] Jourdain G, Chenevert D (2010). Job demands-resources, burnout and intention to leave the nursing profession: a questionnaire survey. Int J Nurs Stud.

[47] National Health Service (2019). NHS Staff Survey Results.

[48] Spiegel DS, Smolen RC, Jonas CK (1986). An examination of the relationships among interpersonal stress, morale and academic performance in male and female medical students. Soc Sci Med.

[49] Anzai E, Douglas C, Bonner A (2014). Nursing practice environment, quality of care, and morale of hospital nurses in Japan. Nurs Health Sci.

[50] Heuser CC, Gibbins KJ, Herrera CA, Thelien LH, Holmgren CM (2018). Moms in medicine: job satisfaction among physician-mothers in obstetrics and gynecology. Work..

[51] Fletcher R, Abel GA, Anderson R, Richards SH, Sailsbury C, Dean SG (2017). Quitting patient care and career break intentions among general practitioners in south West England: findings of a census survey of general practitioners. BMJ Open.

[52] Gilliland AE, Sinclair H, Cuppies ME, McSweeney M, Mac Auley D, O’Dowd TC (1998). Stress and morale in general practice: a comparison of two health care systems. Br J Gen Pr.

[53] Nocon RS, Fairchild PC, Gao Y, Gunter KE, Lee SM, Quinn M (2019). Provider and staff morale, job satisfaction, and burnout over a 4-year medical home intervention. J Gen Intern Med.

[54] McKinstry B, Porter M, Wrate R, Elton R, Shaw J (2004). The MAGPI (morale assessment in general practice index): a new way for doctors to self-assess their morale. Educ Prim Care.

[55] Chandra S, Wright SM, Ghazarian S, Kargul GM, Howell E (2016). Introducing the hospitalist morale index: a new tool that may be relevant for improving provider retention. J Hosp Med.

[56] MacRobert M, Schmele JA, Henson R (1993). An analysis of job morale factors of community health nurses who report a low turnover rate. The research J Nurs Adm.

[57] Yang KP, Huang CK (2005). The effects of staff nurses’ morale on patient satisfaction. J Nurs Res.

[58] Mistry H, Levack WM, Johnson S (2015). Enabling people, not completing tasks: patient perspectives on relationships and staff morale in mental health wards in England. BMC Psychiatry.

[59] Chen JY (2010). Morale and role strain of undergraduate nursing students in a pediatric clinical setting. J Nurs Res..

[60] Spencer K, Foster PE, Whittamore KH, Goldberg SE, Harwood RH (2014). Staff confidence, morale and attitudes in a specialist unit for general hospital patients with dementia and delirium-a qualitative study. Int J Geriatr Psychiatry.

[61] Huby G, Gerry M, McKinstry B, Porter M, Shaw J, Wrate R (2002). Morale among general practitioners: qualitative study exploring relations between partnership arrangements, personal style, and workload. BMJ..

[62] Hartley S, MacFarlane F, Gantley M, Murray E (1999). Influence on general practitioners of teaching undergraduates: qualitative study of London general practitioner teachers. BMJ..

[63] Rucker L, Shapiro J, Fornwalt C, Hundal K, Reddy S, Singson Z (2014). Using focus groups to understand causes for morale decline after introducing change in an IM residency program. BMC Med Educ.

[64] Singh R, Kirtley J, Minhas JS, Lakhani D, Carr S (2019). Exploring junior doctor morale in a UK hospital. J R Coll Physicians Edinb.

[65] Caravella RA, Robinson LA, Wilets I, Weinberg M, Cabaniss DL, Cutler JL (2016). A qualitative study of factors affecting morale in psychiatry residency training. Acad Psychiatry.

[66] Dowling S, Last J, Finnegan H, Daly P, Bourke J, Hanrahan C (2019). Impact of participation in continuing medical education small group learning (CME-SGL) on the stress, morale, and professional isolation of rurally based GPs: a qualitative study in Ireland. BJGP Open.

[67] McKinstry B, Walker J, Porter M, Fulton C, Tait A, Hanley J (2007). The impact of general practitioner morale on patient satisfaction with care: a cross-sectional study. BMC Fam Pr.

[68] McFadzean F, McFadzean E (2005). Riding the emotional roller-coaster: a framework for improving nursing morale. J Health Organ Manag.

[69] Day G, Minichiello V, Madison J (2007). Nursing morale: predictive variables among a sample of registered nurses in Australia. J Nurs Manag.

[70] Cox KB (2001). The effects of unit morale and interpersonal relations on conflict in the nursing unit. J Adv Nurs.

[71] Montoro-Rodriguez J, Small JA (2006). The role of conflict resolution styles on nursing staff morale, burnout, and job satisfaction in long-term care. J Aging Heal.

[72] Barkham M, Richards D, Cahill J, Glanville J, Cooper C, Hardy G (2004). A literature review of staff morale on mental health in-patient units.

[73] Richards DA, Bee P, Barkham M, Gilbody SM, Cahil J, Clanville J (2006). The prevalence of nursing staff stress on adult acute psychiatric in-patient wards. A systematic review. Soc Psychiatry Psychiatr Epidemiol.

[74] Priebe S, Fakhoury WK, Hoffman K, Powell RA (2005). Morale and job perception of community mental health professionals in Berlin and London. Soc Psychiatry Psychiatr Epidemiol.

[75] Galeazzi GM, Delmonte S, Fakhoury W, Priebee S (2004). Morale of mental health professionals in community mental health Services of a Northern Italian Province. Epidemiol Psichiatr Soc.

[76] Johnson S, Osborn DP, Araya R, Wearn E, Paul M, Stafford M (2012). Morale in the English mental health workforce: questionnaire survey. Br J Psychiatry.

[77] Wigley SC (2004). Assessment of morale in further education students studying for A-level examinations. J Furth High Educ.

[78] Nguyen HV, Duong HT, Vu TT (2017). Factors associated with job satisfaction among district hospital health workers in northern Vietnam: a cross-sectional study. Int J Heal Plann Manag.

[79] The World Bank (2019). World Bank Open Data.

[80] Sabitova A, Sajun SZ, Nicholson S, Mosler F, Priebe S (2019). Job morale of physicians in low-income and middle-income countries: a systematic literature review of qualitative studies. BMJ Open.

