# Supplementary material for: Job morale: a scoping review of how the concept developed and is used in healthcare research
Source: BMC Public Health. 2020 Jul 25;20:1166. doi: 10.1186/s12889-020-09256-6 (PMC7382865; doi:10.1186/s12889-020-09256-6)
Supplement: Supplementary file 1 — Additional file 1. Study Characteristics. A table contain study characteristics of all included studies. [file 12889_2020_9256_MOESM1_ESM.docx]

**Additional file 1. Data charting form**

| **N** | **Author(s), year of publication/**  **Country** | **Study design** | **Study context(s)** | **Main objectives** | **Definition(s) of morale (if any)** | **Study population(s)** | **Methodology** | **Outcome measures** | **Main findings** |
| --- | --- | --- | --- | --- | --- | --- | --- | --- | --- |
| 1. | Gal,  1986 (1)/  Israel | Quantitative | Military | To explore the nature, structure and components of the morale construct | “…umbrella term to the cluster of variables pertaining to the military unit’s combat effectiveness” | Israeli Defence Forces | The Combat Readiness Morale Questionnaire – 30 items, 5-point Likert scale | Combat unit morale, cohesion and combat readiness | Variables related to morale: confidence in commanders, team and one’s self, unit cohesion, legitimacy of war and enemy evaluation |
| 2. | Jones, 2006 (2)/  UK | Review | Military | To review the combat experience of British soldier during the First World War | “…high morale would inculcate the necessary offensive spirit” | British soldiers | N/a | N/a | N/a |
| 3. | Manning, 1991 (3)/  USA | Review | Military | To review key concepts, individual and organizational determinants and assessment methods of morale | Adopted Spiegel’s definition of morale: “an unknown quantity, x”, indicating “…the spirit of the army, the grater or less desire to fight and face dangers” | Soldiers | N/a | N/a | Defined individual (fulfilment of basic physical needs, having a goal, role clarity and training) physical and psychological) and organizational (cohesion and esprit de corps) determinants of morale. |
| 4. | Baker, 1930 (4)/  USA | Review | Military and civilian | To explore morale maintenance in military and civilian contexts | Morale “affects individuals in relation to the group” and “it unifies action in relation to the objective of the group;” and “emphasized the spirit of agreement and of co-operative action and is willing to do more than its share without expectation of personal reward” | Soldiers and civilians | N/a | N/a | The importance of leadership was underlined for both military and civilian contexts. |
| 5. | Child,  1941 (5)/  USA | Review | Military and civilian | To perform a bibliographical review of morale concept | Divided morale definitions into three groups (individual, group and individual- within-the group and provided respective definitions | Soldiers and civilians | N/a | N/a | N/a |
| 6. | Park,  1934 (6)/  USA | Review | Industry | To review literature exploring industrial fatigue and group morale | "…a non-logical social code, which regulates relations between persons and their attitudes to one another” | Employees | N/a | N/a | Explained morale as both individual and group phenomenon |
| 7. | Zeleny, 1939 (7)/  USA | Quantitative | Civilian | To measure morale mathematically | “…shared feeling of like” | Civilians | Formulas were proposed to assess morale | Individual and group morale | Individual and group morale can be measured separately |
| 8. | Stuart Chapin and Jahn,  1939 (8)/  USA | Quantitative | Civilian | To explore whether work relief program maintains higher morale than direct relief program | Not provided | Unemployed | Rundquist-Sletto morale scale | Morale in two groups | Difference between morale scores were found |
| 9. | Landis, 1941 (9)/  USA | Review | Civilian | To explore morale and civilian defence | “…a particular state of mind which is shared by members of a group” | N/a | N/a | N/a | Distinction between good and bad morale was made. Several ways to maintain good morale were proposed. |
| 10. | Bateson and Mead,  1941 (10)/  USA | Review | Civilian | To explore principles of morale building | “…high morale is any positive and energetic attitude toward a goal” | N/a | N/a | N/a | Government departments should be oriented towards “discovering, encouraging, and serving local initiative”. |
| 11. | Hocking, 1941 (11)/  USA | Review | Civilian and military | To offer a description and definition of morale | “…state of will, as a man (or group) confronts its objective, a state of ‘willingness’, the mental counterpart of physical fitness or ‘condition’” | N/a | N/a | N/a | The roles of leadership and control of actions were underlined. |
| 12. | Allport and Lepkin, 1943 (12)/  USA | Mixed methods | Civilian | To explore the impact of war news reports on people’s morale in wartime | “the psychological reactions of the citizens” | Civilians | News headlines were offered to citizens and were asked to give their reactions; Likert type survey | Reactions to headlines | The worse the news, the better its morale value (war participation). |
| 13. | Ames, 1941 (13)/  USA | Review | N/a | To explore relationship between morale and religion | “tenacity in the face of adversity” | N/a | N/a | N/a | Religion can strengthen the morale of the nation. |
| 14. | Angell, 1942 (14)/  USA | Review | Civilian | To explore morale from a political perspective | “Morale is both the degree of changed feeling-tone toward the impact of disrupting forces and the capacity to maintain exaltation concerted action” | N/a | N/a | N/a | Role, opportunities and weaknesses of democratic civilian morale were provided. |
| 15. | Hightower, 1944 (15)/  USA | Review | N/a | To provide a sociological conception of morale | Several morale definitions were provided | N/a | N/a | N/a | Morale is a state of institutional or associational health. |
| 16. | Griesser, 1942 (16)/  USA | Review | N/a | To explore underlying factors in democratic morale | Not provided | N/a | N/a | N/a | The importance of leadership and propaganda was highlighted. |
| 17. | Creel,  1941 (17)/  USA | Review | Civilian | To explore propaganda as a tool to maintain morale | Not provided | N/a | N/a | N/a | The formation of civilian morale should be done through truthful information. |
| 18. | Anderson,  1943 (18)/  USA | Review | Civilian | To explore effects of food rationing on morale | Morale can be characterised by the presence of common values, motivation and consistency. | N/a | N/a | N/a | Rationing effects the belief in common values; affects the determination to cooperate; it strengthens and weakens the degree of consistency between the values of individuals and the group. |
| 19. | Woods, 1946 (19)/  USA | Observational | Civilians | To explore morale among raw recruits | “...a term used to describe special effort or willingness to make extreme effort or sacrifice for the purpose of securing some socially recognized value of fact” | Recruits | Each participant was observed during different activities | Participants with high, low and indifferent morale | Civilian values influence military morale; there are factors influencing morale; the degree of morale may vary across individuals. |
| 20. | Durant, 1941 (20)/  USA | Review | N/a | To explore morale measurement methods | “Morale is the relationship of a group” | N/a | N/a | N/a | Factors influencing morale vary from country to country. Measurable indices of civilian morale can be found for each country and used. |
| 21. | Shils,  1941 (21)/  USA | Review | Civilians | To review governmental research on attitudes and morale | Not provided | N/a | N/a | N/a | Analysis of complaint letters; qualitative interviews; analysis of frequency of civil strikes. |
| 22. | Estorick, 1941 (22)/  UK | Review | Civilian and military | To review morale in contemporary England | “…a state of abundant psychosomatic health marked subjectively by an energetic, decisive resolution to achieve a given goal and objectively by spirited, unyielding, co-operative, or co- ordinated efforts in the direction of that goal; or, lacking proper stimulus, morale is a state of readiness for such determination and such efforts.” | N/a | N/a | N/a | Morale in England changed alongside with major social changes. |
| 23. | Wirth,  1941 (23)/  USA | Review | Minority groups | To explore morale of minority groups | “… element in collective action which enables the participants to persist in their determination to achieve their collective purpose” | N/a | N/a | N/a | Morale-building should be based on the diversity of origin of citizens. |
| 24. | Park,  1941 (24)/  USA | Review | Civilian and military | To explore effect of propaganda on morale | “will to fight”; “the ability to endure hardships at home and bad news from the front” | N/a | N/a | N/a | Propaganda is crucial in forming public opinion and morale. |
| 25. | MacRury, 1949 (25)/  USA | Review | Industry | To review methods of assessing employee morale | Absence records are “invaluable indications of the status of employee morale” | Employees | Survey | Absence records and opinion polls | Absence records and opinion polls can be used as indicators of employee morale |
| 26. | Webb and Hollander, 1956 (26)/  USA | Quantitative | Aviation | To compare three morale measures: a survey, pooled group judgments, and self-evaluations | “…an interest and enthusiasm for the naval air program” | Air program trainees | Survey | Morale index | Peer nominations and direct self-descriptions may have greater utility than morale index. |
| 27. | Guion,  1958 (27)/  USA | Qualitative | Industry | To explore the problem of morale terminology | “Morale is the extent to which an individual’s needs are satisfied and the extent to which the individual perceives that satisfaction as steaming from his total job situation” | Not provided | Definitions of morale were collected | Definitions of morale | Definition was proposed |
| 28. | Scott and Rowland, 1970 (28)/  USA | Quantitative | Industry | To investigate the generality of the factors across samples from different organizations | “… a multidimensional construct, encompassing both affective and cognitive components” | Civil service employees | Surveys | ‘Me at work’ concept | Factors were congruent across both samples |
| 29. | Giese, 1949 (29)/  USA | Quantitative | Industry | To analyse the relationship of the objective records of departmental performances to morale | Not provided | Department store employees | Morale questionnaire | Six morale factors were considered | Limitations of the morale survey were stated (costly, prevents the detection of an undesirable trend in morale) |
| 30. | Goode and Fowler, 1949 (30)/  USA | Review | Industry | To analyse incentive factors in a low morale plant | Not provided | Automobile industry employees | N/a | N/a | Goals should be clearly defined and be simple; skills should be well-known; functional roles should be clear |
| 31. | Browne and Neitzel, 1952 (31)/  USA | Quantitative | Industry | To explore relationships between communication, supervision and morale | Not provided | Utilities company employees | Morale scale by Harris - 31 items | Morale score | Negative association between morale and disparity scores was found; positive association between morale and the echelon level of the supervisors was found. |
| 32. | Blocker and Richardson, 1963 (32)/  USA | Review | Education | To review morale research | Not provided | Teachers | N/a | N/a | Noted that: terms morale and job satisfaction were used interchangeably; morale does not have validated scale; scale should be supported by qualitative investigation. |
| 33. | Campbell and Tyler, 1957 (33)/  USA | Mixed | Industry | To review the validity of morale surveys | Not provided | Farm Bureau Insurance Companies employees; submarines employees. | Survey and open questions | Group morale score | The validity of morale surveys was proved |
| 34. | Baehr and Renck,  1958 (34)/  USA | Review | Industry | To suggest factors underlying employee morale | Morale was defined using 4 main approaches: needs psychology, hierarchy of needs, the significance of interactions among members of the group; theory of motivation. The employee morale inventory was invented. | Industry employees | Employee inventory | Factors underlying morale | Main factors: organisation and management; immediate supervision; material rewards; fellow employees; job satisfaction |
| 35. | Worthy, 1950 (35)/  USA | Mixed | Industry | To assist executives in their efforts to maintain sound and mutually satisfactory employee relationships | Not provided | Company employees | Morale survey and qualitative interviews were integrated | “feeling tone’ of employees with respect to six key areas | Used high and low terminology. Morale deterioration was related to two trends: increasing size of the administrative unit; increasing complexity of organizational structure |
| 36. | Herzberg,  1954 (36)/  USA | Review | Industry | To analyse morale survey | Not provided | Power and steel companies’ employees | Morale booklet – survey and open questions | Survey items and comments were divided into favourable and unfavourable | The majority feeling was favourable toward a job. Comments were meaningful and provide specific information to clarify morale; unfavourable comments were more specific. |
| 37. | Payne et al.,  1976 (37)/  UK | Review | N/a | To clarify the meaning and measurement of the concepts of organizational climate and job satisfaction | Not provided | N/a | N/a | N/a | Noted that job satisfaction and morale are not the same, yet related. Difference between job/role morale and organizational morale. |
| 38. | Organ and Ryan,  1995 (38)/  USA | Review | N/a | To explore relationship between job satisfaction and organizational citizenship behaviour | Morale was perceived as an underlying value ‘m’ | N/a | Quantitative review of 55 studies | N/a | Morale is taken to be a basic psychological factor ‘m’ that influences attitudinal measures |
| 39. | Vandenberg and Nelson/ 1999 (39)/  USA | Quantitative | Industry | To explore the variability in the intention-behaviour relationship | Not provided | Multinational firm employees | 3 groups of participants a set of scales | Perceived employment opportunities; job satisfaction; supervisory motives; organizational motives; locus of control | An idea that there is a unifying variable for job satisfaction, organizational motives and turnover intentions was proposed |
| 40. | McKnight et al., 2001 (40)/  Italy, Japan, USA | Mixed | Industry | To test the moderating and direct effect of employee-management relationship closeness | “…the degree to which an employee feels good about his or her work and work environment” | Computer operations professionals | Qualitative interviews; survey | Relationship between employee-manager | Developed a conceptual model of employee morale based on qualitative interviews. Morale was used a term encompassing intrinsic motivation, job satisfaction, work meaningfulness, organizational commitment and pride in one’s work. |
| 41. | Weakliem and Frenkel, 2006 (41)/  Australia | Quantitative | Not provided | To consider the relationships between morale and workplace productivity | “prescribed activities of the group” | Sample of Australian workplaces with more than 20 employees | Australian workplace industrial relations survey | Productivity; morale and control variables (age, size of workplace, number of unions and others) | Morale influences productivity in a linear fashion. Effect is larger when management regards product quality as important and attempts to develop a corporate ethic and culture. Morale was associated with greater work effort. |
| 42. | Motowidlo and Borman,  1978 (42)/  USA | Quantitative | Military | To explore relationships between military morale, motivation, satisfaction and unit effectiveness | “It is more general than concepts of motivation and satisfaction that appear in the psychology literature and seems to encompass major elements of both concepts as well as the notion of group cohesiveness.” | Soldiers | Behavioural morale scales; self-rated motivation and satisfaction | Morale; motivation; satisfaction; | Ratings of platoon morale correlated most strongly with mean self-reports of *overall* satisfaction and somewhat less strongly with self-reports of satisfaction with narrower facets of Army life. |
| 43. | Abbott,  2003 (43)/  UK | Review | Industry | To explore whether employee satisfaction affects profit | Not provided | Focus industries were technology, processing and business banking | N/a | Employee/ customer satisfaction | Morale can be low, yet employees work hard to increase customer satisfaction and maximise company profits. |
| 44. | Johnsrud et al.,  2000 (44)/  USA | Quantitative | Education | To define the construct of morale and investigate its validity | “…’umbrella’ notion that includes, in addition to satisfaction with the work environment, such attributes as enthusiasm, commitment or loyalty to the institution, willingness to work, and dedication to common goals” | Midlevel administrators within a ten-campus university system | Likert scales | Perceptions of work life; overall morale; intent to leave | Multilevel theoretical model was proposed, defining morale as a multidimensional construct comprised of institutional regard (care, fare, empvalue); quality of work (variety, purpose, freedom, satisfaction) and mutual loyalty (loyal, orgvalue). |
| 45. | Armstrong-Stassen et al.,  2004 (45)/  Canada | Quantitative | Civil service | To investigate the interactive effect of work-group membership stability and time on survivors’ reactions to organizational downsizing | Not provided | Non-management employees of a federal government department | Likert scale | Job-related factors (satisfaction, involvement, performance, workload, perceived justice); group-related factors; work-group membership | Survivors in moderate-change work groups reported an increase in job satisfaction, job security, job performance and employee morale. |
| 46. | Kandori,  2003 (46)/  Japan | Quantitative | N/a | To examine why the performance of an organization is often subject to gradual erosion | Not provided | N/a | Mathematical modelling | N/a | Certain norms and morale are sustainable and do not necessarily depend on material incentives. |
| 47. | Kennedy,  1995 (47)/  Canada | Quantitative | N/a | To incorporate the notion of worker morale into an economic model of pay and performance and examine its implications | ‘team spirit’ | N/a | Mathematical modelling | N/a | A profit-sharing component reduces the pay differential and boost morale, improve productivity and profitability |
| 48. | Peterson et al.,  2008 (48)/  USA | Review | N/a | To suggest placing morale under the positive psychology umbrella | “as an indicator of group well-being, just as life satisfaction is an indicator of individual well-being” | N/a | N/a | N/a | The components of morale were suggested. |
| 49. | Britt et al., 2007 (49)/  USA | Quantitative | Military | To explore correlates and consequences of morale versus depression under stressful conditions | “… motivation and enthusiasm to perform well within a specified context (such as a military operation)”. Morale should be measured with a referent (in a particular field). | Soldiers | Data from longitudinal study was used for structural equation modelling. Four-itemed Likert scale was used measure morale. | Morale score; depression; engagement in meaningful work; task significance; confidence in unit functioning and leadership. | Morale was predicted by indices of engagement in meaningful work and confidence in unit functioning and leadership. Morale should measure with a referent. |
| 50. | Evans,  2001 (50)/  UK | Mixed | Education | To identify and examine factors which influence teachers morale and job satisfaction | “… a state of mind encompassing all of the feelings determined by the individual’s anticipation of the extent of satisfaction of those needs which s/he perceives as significantly affecting his/her total work situation”. | Schoolteachers and academics | Multi-staged study: observational; survey; qualitative interviews. | Morale, job satisfaction, motivation | Levels, key factors, general sources of job motivation, job satisfaction and morale were proposed. ‘Person-organization fit’ features were defined. |
| 51. | Hart,  1994 (51)/  Australia | Quantitative | Education | To investigate the relationship between teachers’ negative work experiences and levels of distress and morale | “…the degree of energy, enthusiasm, team spirit and pride that teachers perceived in their school” | Primary and secondary school teachers | Morale subscale of School Organizational Health Questionnaire; The General Strain Index | Psychological distress; morale | Psychological distress and morale are separate outcomes of positive and negative work experiences; both contributed to teachers’ quality of work life. |
| 52. | Gulliver et al.,  2003 (52)/  UK | Quantitative | Healthcare | to apply the Bedian and Armenakis model to aid in the explanation of changes in job satisfaction and morale during an integration of mental health and social services | Not provided | Mental healthcare professionals | Theoretical framework by Bedian and Armenakis was employed. The Likert survey developed by Onyett was used. | Role clarity, team identification, professional identification; job satisfaction; burnout. | One year after integration, there was a significant decrease in the mean level of role clarity and job satisfaction; an increase in mean reported experiences of emotional exhaustion. |
| 53. | Johnson et al.,  2011 (53)/  UK | Mixed (multi-staged) – reported in several papers | Healthcare | To examine the morale of the National Healthcare service (NHS inpatient mental health workforce | “…a general term encompassing the main aspects of work-related well-being and satisfaction and engagement with work” | Mental healthcare professionals | Warr’s framework of affective well-being was employed to define morale dimensions. Also, Karaseks’ Job Demand-Control Model was used to encompass factors influencing job morale. | Five measures: burnout, well-being, overall psychological health, job satisfaction, job motivation. | NHS inpatient mental health staff reported good job satisfaction and a sense of achievement from their work. Individual-level factors were found to be more important. Job Demand-Control Model was largely upheld. Additionally, organisational context, adverse incidents, staff perception of the quality of the ward environment were associated with morale. |
| 54. | Totman et al.,  2011 (54)/  UK | Qualitative | Healthcare | To explore mechanisms underlying good and poor morale. | Not provided | Mental healthcare professionals | Individual and group interviews | Views about good and poor morale | Good morale was sustained by mutual loyalty and trust within cohesive teams; clear roles, supportive ward managers, well designed organisational procedures and structures. Morale threats: insufficient staffing levels. Are not safe and don’t have enough time to spend with patients; the high risk of violence and lack of voice. |
| 55. | Johnson et al.,  2012 (55)/  UK | Quantitative | Healthcare | To describe staff well-being and satisfaction in a multicentre UK NHS sample and explore associated factors | “…a general term encompassing the main aspects of work-related well-being and satisfaction and engagement with work” | Mental healthcare professionals | Warr’s framework of affective well-being was employed to define morale dimensions. Also, Karaseks’ Job Demand-Control Model was used to encompass factors influencing job morale. | Five measures: burnout, well-being, overall psychological health, job satisfaction, job motivation. | Well-being and job satisfaction were good, yet emotional exhaustion was high. All morale indicators were intercorrelated. |
| 56. | Mistry et al.,  2015 (56)/  UK | Qualitative | Healthcare | To explore staff morale and staff-patient relationships from a patient perspective | Not provided | Patients of inpatient wards | Individual interviews | Views about staff well-being and morale | Patients valued staff who worked as a cohesive team, practised in a collaborative way and used enabling approaches to support their recovery. |
| 57. | Napier and Clinch,  2019 (57)/  UK | Qualitative | Healthcare | To explore the impact upon morale and retirement decisions of changes in psychological aspects of UK general practice over the course of a career | Not provided | GPs | Biographical narrative interviewing method. Job Demands-Control-Support (JDCS) model was applied. | Views on institutional and social changes affecting morale | Changes in the psychological work environment; reduction in autonomy; increasing demands; fragmented teams contributed to decline in morale. JDCS model was tested and can be used to inform policies. |
| 58. | Wang et al., 2018 (58)/  Taiwan | Quantitative | Healthcare | To determine the influencing factors on morale and developing a structural model of morale | Not provided | Certified nursing assistants | Theoretical model was constructed and tested (altruistic personality scale; inventory of socially supportive behaviours; Collect-Lester fear of death scale; job satisfaction scale; stress checklist; employee morale questionnaire. | Altruism; social support; death perception; job satisfaction; job stress; morale. | Five constructs to morale  (5CM) model was developed and tested. The relationship between altruism and morale was the strongest; job stress was the major mediator. |
| 59. | Nguyen et al., 2017 (59)/  Vietnam | Review | Healthcare | To systematically collate effort-reward imbalance rates among healthcare workers | Not provided | Healthcare workers | Systematic review methodology. | Extrinsic effort; reward | Healthcare workers contributed more than they are rewarded especially in Japan, Vietnam, Greece and Germany. |
| 60. | McVicar,  2016 (60)/  UK | Review | Healthcare | To identify core antecedents of job stress and job satisfaction | Not provided | Nurses | Comparative scoping review methodology. Job demands resources model was applied. | Job stress; job satisfaction | Antecedents: emotional demands; changing work environment; shift work. |
| 61. | Turnell et al.,  2016 (61)/  Australia | Quantitative | Healthcare | To examine the prevalence and predictors of burnout and work engagement | Not provided | Psychosocial-oncologist clinicians | Maslach burnout inventory; Utrecht work engagement scale | Burnout; work engagement; measures of job demands and resources | Lower levels of job resources and higher levels of job demands predicted higher burnout. Higher job resources predicted higher levels of work engagement. |
| 62. | Spence Laschinger et al.,  2012 (62)/  Canada | Quantitative | Healthcare | To test the job demands-resources model among nurses | Not provided | New graduate nurses | Worklife scale; Negative acts questionnaire revised; Psychological capital questionnaire; Maslach burnout inventory | Job demands; job resources; burnout | Job demands (workload and bullying) predicted burnout; job resources (supportive environment and control) predicted work engagement. |
| 63. | Jourdain and Chenevert,  2010 (63)/  Canada | Quantitative | Healthcare | To investigate the. Nature of the relationship between burnout and intention to leave | Not provided | Nurses | Quantitative overload scale; psychological empowerment; burnout | Job demands; job resources; burnout | Demands are he most important determinant of emotional exhaustion; resources predicted depersonalization. |
| 64. | Heuser at al.,  2017 (64)/  USA | Quantitative | Healthcare | To define factors that affect physician satisfaction | Not provided | Physician-mothers in obstetrics and gynaecology | Self-developed survey. Morale was measured by rating professional morale. | Work/life satisfaction | Majority rated professional morale as very/somewhat positive. Participants were dissatisfied with time for extra-professional activities. |
| 65. | Spiegel et al.,  1986 (65)/  USA | Quantitative | Healthcare | To examine the relationships among interpersonal stress, morale and academic performance | Not provided | Medical students | Conflict situation inventory; morale was measured by single item Likert scale; data on academic performance. | Interpersonal stress; morale; academic performance. | Morale and interpersonal stress predicted academic performance. Inverse relationship between morale and interpersonal stress was stronger for females. |
| 66. | Anzai et al.,  2014 (66)/  Japan | Quantitative | Healthcare | To describe perceptions of the nursing practice environment and examine its associations | Not provided | Nurses | Practice environment scale; ability to provide quality of nursing care; quality of care; ward morale – single item Likert scales. | Practice environment; quality of care; ward morale. | Nursing practice environment was a significant predictor of patient and organizational outcomes. |
| 67. | Cox,  2001 (67)/  USA | Quantitative | Healthcare | To explore the effects of unit morale and interpersonal relations on conflict in the nursing unit | Not provided | Nurses | Cox conflict scale; unit technology scale; work satisfaction scale; effectiveness of team performance; anticipated turnover scale. | Unit technology;  Intragroup conflict; work satisfaction; team effectiveness; turnover | Unit morale and interpersonal relations dimension of team performance effectiveness was negatively associated with intragroup conflict and anticipated turnover, and positively associated with satisfaction with pay. |
| 68. | Fletcher et al.,  2017 (68)/  UK | Quantitative | Healthcare | To describe GPs career intentions | Not provided | GPs | Self-developed questionnaire. Morale was measured by single item Likert scale. | Career intentions | Majority reported low morale. Morale predicted career intentions. |
| 69. | Gilliland et al.,  1998 (69)/  Northern Ireland (NI) and Republic of Ireland (ROI) | Quantitative | Healthcare | To compare perceived levels of stress and morale between GPs working in two different countries | “…feeling of confidence in one’s situation with a positive hope for the future” | GPs | Self-developed questionnaire. Morale was measured by single item Likert scale. | Stress; morale | NI had higher stress and lower morale than in the ROI. |
| 70. | Nocon et al.,  2019 (70)/  USA | Quantitative | Healthcare | To determine how morale, job satisfaction and burnout changed over time | Not provided | Primary care providers | Self-developed questionnaire. Morale was measured by single item Likert scale. | Morale; job satisfaction; burnout. | Job satisfaction decreased, whereas burnout increased. |
| 71. | McKinstry et al.,  2004 (71)/  UK | Quantitative | Healthcare | To design a questionnaire for GPs to detect morale | Not provided | GPs | MAGPI was compared with the Global Health Questionnaire | N/a | Morale Assessment in General Practice Index (MAGPI) was developed |
| 72. | Chandra et al.,  2016 (72)/  USA | Quantitative | Healthcare | To develop a valid instrument to assess morale and explore the relationship between morale and intention to leave | “…the emotional or mental condition with respect to cheerfulness, confidence, or zeal and is especially relevant in the face of opposition or hardship” | Hospitalists | Confirmatory factor analysis | N/a | Hospitalist Morale Index (HMI) was developed. |
| 73. | MacRobert et al.,  1993 (73)/  USA | Quantitative | Healthcare | To define positive retention factors | “Morale as a retention factor” | Nurses | The Science Research associates morale inventory (14 items) | Morale, demographic variables | Nurses with advanced education preparation, higher positions demonstrated increased level of morale. |
| 74. | Yang and Huang,  2005 (74)/  Taiwan | Quantitative | Healthcare | To examine staff nurses’ morale and its effect on patient satisfaction | Not provided | Nurses | Work Morale Scale; Nursing-Sensitive Patient Satisfaction Scale (16 items) | Job involvement; organizational identification; group cohesiveness. | Job position and pay had a significant effect on nurses’ work morale. |
| 75. | Grieve,  1997 (75)/  UK | Quantitative | Healthcare | To devise and apply a measure of mental well-being in general practitioners, and to use this to investigate the effect of practice are deprivation | Well-being was used to explore morale | GPs | Self-developed questionnaire | Well-being; workload; burnout | Well-being was not associated with practice area deprivation. |
| 76. | Galeazzi et al., 2004 (76)/  Italy | Mixed | Healthcare | To explore morale of psychiatrists and psychiatric nurses | Morale was perceived as an ‘umbrella’ term | Mental health professionals | Team Identity Scale; Minnesota Job Satisfaction Scale; Maslach Burnout Inventory; open questions. | Burnout; team identity; job satisfaction. | Psychiatrists had higher scores o |
| 77. | Priebe et al.,  2005 (77)/  Germany and UK | Mixed | Healthcare | To assess morale and compare findings between the groups | Morale was perceived as an ‘umbrella’ term | Mental health professionals | Team Identity Scale; Minnesota Job Satisfaction Scale; Maslach Burnout Inventory; open questions. | Burnout; team identity; job satisfaction. | Working in London predicted higher burnout, lower job satisfaction and lower team identity. Being a psychiatrist predicted higher team identity. |
| 78. | Reininghaus and Priebe,  2007 (78)/  UK, Austria, Germany, Italy | Review | Healthcare | To assess the extent to which levels of morale are sensitive to differences between professional groups and healthcare systems, and identify factors influencing morale | Morale was perceived as an ‘umbrella’ term | Mental health professionals | Team Identity Scale; Minnesota Job Satisfaction Scale; Maslach Burnout Inventory. | Pooled analysis methodology. Burnout; team identity; job satisfaction. | Staff in community mental healthcare show substantial differences in morale levels depending on the professional group and context. |
| 79. | Bowers et al.,  2008 (79)/  UK | Quantitative | Healthcare | To assess the relationship of staff morale to patient, service environment and etc. | “…morale is a broad and complex concept which is generally taken to be a composite if low stress, low burnout, high job satisfaction, low staff sickness, low absenteeism and low staff turnover”. | Mental health professionals | Maslach Burnout Inventory | Burnout is taken as a proxy measure, as the MBI correlates with many other components of morale. | Morale was higher than published comparison samples. Length of time in post was correlated with low morale, and qualified nurses had higher emotional exhaustion and personal accomplishment. |
| 80. | Caravella et al.,  2016 (80)/  USA | Qualitative | Healthcare | To identify important factors affecting morale in psychiatry residency training | “… burnout, individual mental health and wellness and job satisfaction would be important factors determining individual and group morale” | Psychiatry residents | Semi-structured focus groups | Views on positive and negative morale factors | Factors related to sense of community and individual motivators enhanced resident morale. |
| 81. | Chen,  2010 (81)/  Taiwan | Qualitative | Healthcare | To examine the morale and the potential role strains of undergraduate nursing students | “Morale is the mental and emotional condition of a person or a group with regard to its function, which is exhibited by a disposition marked by confidence, cheerfulness, discipline, and willingness that drives the individual desire to success” | Nursing students | Group interviews | Views on morale | Various clinical settings, individual perceptions and capacities, and conflicts between expectative and ideal roles were found to induce morale reaction and role strain in nursing students. |
| 82. | Spencer et al.,  2014 (82)/  UK | Qualitative | Healthcare | To explore confidence, morale and attitudes amongst staff | Not provided | Mental health professionals | Individual interviews | Views on morale | Confidence, team spirit, training, supportive culture improved morale. |
| 83. | Huby et al.,  2002 (83)/  UK | Qualitative | Healthcare | To explore GPs experiences of well-being and distress at work | Not provided | GPs | Unstructured interviews; guided interviews; focus groups. | Experience of well-being and distress at work | Key factors were defined: workload; personal style; practice arrangement. |
| 84. | Hartley et al.,  1999 (84)/  UK | Qualitative | Healthcare | To examine the perceived effect of teaching clinical skills and associated teacher training programmes on GPs morale | Not provided | GPs | Semi-structured interviews | Views on morale | Teaching clinical skills have positive effect on GPs morale. |
| 85. | Rucker et al.,  2014 (85)/  USA | Qualitative | Healthcare | To identify systemic problems that result in declines in morale | Not provided | Medicine residents | Focus groups | Views on reasons for deteriorating morale | Major factors contributing to deteriorating morale: pace of change; process of change; the role of chief residents in change; fear of intimidation. |
| 86. | Singh et al.,  2019 (86)/  UK | Mixed | Healthcare | To explore the morale of junior doctors and improve understanding of the major contributing factors | Not provided | Junior doctors | Online survey and open questions | Overall morale and contributing factors | Factors contributing positively on morale: feeling part of a team; being recognised for good practice; being able to take regular breaks; ease of opportunity to take leave for personal life and events; having access to a computer or workstation. |
| 87. | Dowling et al.,  2019 (87)/  Ireland | Qualitative | Healthcare | To examine the effectiveness of regular participation in continuing medical education (CME) | Not provided | GPs | Semi-structured focus groups | The impact of CME | Regular meetings with an established group of trusted colleagues provided a ‘safe space’, which boosted morale. |
| 88. | McKinstry et al.,  2007 (88)/  UK | Quantitative | Healthcare | To examine the impact of GPs morale on patient satisfaction | Not provided | GPs | Morale assessment in general practice index (MAGPI); General practice assessment questionnaire (GPAQ) | MAGPI score | No relationship between overall GP morale and patient perception of performance. |
| 89. | Montoro-Rodriguez and Small,  2006 (89)/  Canada | Quantitative | Healthcare | To examine the relationships between individual characteristics, work demands, work resources, and conflict resolution strategies on the one hand and nursing staff outcomes such as psychological morale, burnout and satisfaction | Literature on morale was reviewed and several understandings of morale | Nurses | The model of stress by Ramirez et al. (1998) and Cohen-Mansfield and Noelker (2000) was applied. Shortened version of the Psychiatric epidemiology research instrument; Maslach burnout inventory; Job satisfaction scale. | Staff morale; burnout; job satisfaction | Nurses’ psychological morale, occupational stress, and job satisfaction are influenced by conflict resolution styles, after controlling by individual characteristics, work demands, and work resources factors. |
| 90. | McFadzean and McFadzean,  2005 (90)/  UK | Review | Healthcare | To examine the literature on employee morale and to construct a model of this area pertaining to nurses | “Morale is the degree to which an employee exhibits a positive or motivated psychological state. It can manifest itself as pride in the organisation and its goals, faith in its leadership, and a sense of shared purpose with, and loyalty to, others in the organisation.” | Nurses | Scoping review methodology | N/a | A framework for improving nursing morale was developed. |
| 91. | Day et al.,  2006 (91)/  Australia | Review | Healthcare | To review the literature surrounding nursing morale and the variables impacting it. | It has been noted that there has been no unifying morale definition. | Nurses | Scoping review methodology | N/a | Low morale is costly to organisations and it is a key source of poor patient outcomes and care. |
| 92. | Day et al.,  2007 (92)/  Australia | Quantitative | Healthcare | To investigate the determinants of morale among nurses | Not provided | Nurses | 160-itemed survey. Morale was measured by 4-point Likert scale. | Abuse; professional autonomy; work climate; organizational morale; personal morale. | Organizational morale was associated with patient care provision and team interaction. Personal morale was positively associated with team interaction, consultation, professional recognition and lower levels of patient abuse. |
| 93. | Callaghan,  2003 (93)/  UK | Qualitative | Healthcare | To explore nursing morale and determine the factors influencing it | Not provided | Nurses | Individua in-depth interviews | Views about morale and their concerns about their career. | Morale was low. A large number of nurses were considering leaving the profession. Negative factors were low pay, lack of support, limited opportunity for promotion, lack of resources. |
| 94. | Richards et al.,  2006 (94)/  UK | Review | Healthcare | To review the prevalence of low staff morale, stress, burnout, job satisfaction and psychological wellbeing among staff working in in-patient psychiatric wards. | Morale was measures by a set of indicators | Staff in in-patient mental healthcare settings | Systematic review methodology | Indicators of poor staff morale: occupational stress, job satisfaction, burnout, psychological well-being and the incidence of psychiatric disorders, staff absenteeism, recruitment and retention issues. | Most studies did not find very high levels of staff burnout and poor morale but were small and quality of poor. |
| 95. | Cahill et al.,  2004 (95)/  UK | Review | Healthcare | To examine the extent, aetiology and consequences of poor staff morale in inpatient mental health services, and to identify the clinical and cost effectiveness of strategies to improve morale | Morale was measures by a set of indicators | Staff in in-patient mental healthcare settings | Systematic review methodology | Indicators of morale: occupational stress, job satisfaction, burnout, mental well-being, the incidence of psychiatric disorders, staff sickness, staff absenteeism, recruitment and retention of staff. | Heterogeneity of primary outcomes, measurement instruments. The small amount of data is available suggests that levels of burnout is moderate, job satisfaction is high. Factors: organisational issues and psychological variables. Interventions: educational, too little evidence on psychological and structural interventions. |
| 96. | Gilbody et al.,  2009 (96)/  UK | Review | Healthcare | To examine the impact and cost effectiveness of strategies to improve staff morale and reduce burnout among staff working in psychiatric units | Morale was measures by a set of indicators | Staff working in psychiatric units | Systematic review methodology | Indicators of morale: psychological well-being, job satisfaction, burnout, stress, staff sickness and turnover. | Strategies: educational, phyco-social, organisational. |

**References**

1. Gal R. Unit morale: From a theoretical puzzle to an empirical illustration: An Israeli example. Journal of Applied Social Psychology. 1986;16(6):549-64.

2. Jones E. The Psychology of Killing: The Combat Experience of British Soldiers during the First World War. Journal of Contemporary History. 2016; 41 (2). 229-46.

3. Manning FJ. Morale, cohesion, and esprit de corps. In: Gal R, Mangelsdorff AD, editors. Handbook of military psychology. Oxford, England: John Wiley & Sons; 1991. p.453-70.

4. Baker HJ. The Maintenance of Morale. The International Journal of Ethics. 1930;40(4):542-9.

5. L. Child I. Morale: a bibliographical review. Psychol Bull. 1941; 38(6): 393-420.

6. Park RE. Industrial Fatigue and Group Morale. Am J Sociol. 1934;40(3):349-56.

7. Zeleny LD. Sociometry of Morale. American Sociological Review. 1939;4(6):799-808.

8. Stuart Chapin F, Jahn JA. The Advantages of Work Relief Over Direct Relief in Maintaining Morale in St. Paul in 1939. Am J Sociology. 1940; 46(1):13-22.

9. Landis JM. Morale and Civilian Defense. Am J Sociol. 1941;47(3):331-9.

10. Bateson G, Mead M. Principles of Moral Building. The Journal of Educational Sociology. 1941;15(4):206-20.

11. Hocking WE. The Nature of Morale. Am J Sociol. 1941;47(3):302-20.

12. Allport FH, Lepkin M. Building War Morale with News-Headlines. The Public Opinion Quarterly. 1943;7(2):211-21.

13. Ames ES. Morale and Religion. Am J Sociol. 1941;47(3):384-93.

14. Angell E. Civilian Morale: Democracy's New Line of Battle. The Journal of Educational Sociology. 1942;15(7):383-93.

15. Hightower RL. A Sociological Conception of Morale. Social Forces. 1944;22(4):410-5.

16. Griesser M. Underlying Factors in Democratic Morale. The Journal of Educational Sociology. 1942;15(7):414-20.

17. Creel G. Propaganda and Morale. Am J Sociol. 1941;47(3):340-51.

18. Anderson CA. Food Rationing and Morale. American Sociological Review. 1943;8(1):23-33.

19. Montgomery, Woods WA, Sadler WS, Jr., Benge EJ, Warner N. Morale: with particular reference to the British soldier. Med Press. 1946;216(19):351-4.

20. Durant H. Morale and Its Measurement. Am J Sociol. 1941;47(3):406-14.

21. Shils EA. A Note on Governmental Research on Attitudes and Morale. Am J Sociol. 1941;47(3):472-80.

22. Estorick E. Morale in Contemporary England. Am J Sociol. 1941;47(3):462-71.

23. Wirth L. Morale and Minority Groups. Am J Sociol. 1941;47(3):415-33.

24. Park RE. Morale and the News. Am J Sociol. 1941;47(3):360-77.

25. MacRury K. Employee Morale: Analyses of Absence Records and Opinion Polls. Industrial and Labor Relations Review. 1949;2(2):237-47.

26. Webb WB, Hollander EP. Comparison of three morale measures: a survey, pooled group judgments, and self-evaluations. Journal of Applied Psychology. 1956;40(1):17-20.

27. Guion R. The problem of Terminology in Industrial Morale. (A Symposium). Personnel Psychology. 1958;11:59-64.

28. Scott WE, Rowland KM. The generality and significance of semantic differential scales as measures of “morale”. Organizational Behavior and Human Performance. 1970;5(6):576-91.

29. Giese WJ, Ruter HW. An objective analysis of morale. The Journal of applied psychology. 1949;33(5):421-7.

30. Goode WJ, Fowler I. Incentive Factors in a Low Morale Plant. American Sociological Review. 1949;14(5):618-24.

31. Browne CG, Neitzel BJ. Communication, Supervision, and Morale. Journal of Applied Psychology. 1952;26(2):86-91.

32. Blocker CE, Richardson R. Twenty-Five Years of Morale Research: A Critical Review. 1963. 200-10.

33. Campbell DT, Tyler BB. The Construct Validity of Work-Group Morale Measures. Journal of Applied Psychology. 1957;41(2):91-2.

34. Baehr ME, Renck R. The Definition and Measurement of Employee Morale. Administrative Science Quarterly. 1958;3(2):157-84.

35. Worthy JC. Organizational Structure and Employe Morale. American Sociological Review. 1950;15(2):169-79.

36. Herzberg F. An Analysis of Morale Survey Comments. Personnel Psychology. 1954;7(2):267-75.

37. Payne RL, Fineman S, Wall TD. Organizational climate and job satisfaction: a conceptual synthesis. Organizational Behavior and Human Performance. 1976;16(1):45-62.

38. Organ DW, Ryan K. A meta-analytic review of attitudinal and dispositional predictors of organizational citizenship behavior Personnel Psychology. 1995;48(4):775-802.

39. Vandenberg RJ, Nelson JB. Disaggregating the Motives Underlying Tiarnover Intentions: When Do Intentions Predict Turnover Behavior? Human Relations. 1999;52(10):1313-36.

40. McKnight D, Ahmad S, Schroeder R. When do Feedback, Incentive Control, and Autonomy Improve Morale? The Importance of Employee-Management Relationship Closeness. Journal of Managerial Issues. 2001;13(4): 466-82.

41. Weakliem DL, Frenkel SJ. Morale and workplace performance. Work and Occupations. 2006;33(3):335-61.

42. Motowidlo SJ, Borman WC. Relationships between military morale, motivation, satisfaction, and unit effectiveness. Journal of Applied Psychology. 1978;63(1):47-52.

43. Abbott J. Does employee satisfaction matter? A study to determine whether low employee morale affects customer satisfaction and profits in the business-to-business sector. Journal of Communication Management. 2003;7:333-9.

44. Johnsrud LK, Heck RH, Rosser VJ. Morale Matters: Midlevel Administrators and Their Intent to Leave. The Journal of Higher Education. 2000;71(1):34-59.

45. Armstrong-Stassen M, Wagar T, Cattaneo R. Work‐Group Membership (In)Stability and Survivors' Reactions to Organizational Downsizing. Journal of Applied Social Psychology. 2006;34:2023-44.

46. Kandori M. The Erosion and Sustainability of Norms and Morale. Japanese Economic Review. 2003;54:29-48.

47. Kennedy PW. Performance Pay, Productivity and Morale. Economic Record. 1995;71(3); 240-47.

48. Peterson C, Park N, Sweeney PJ. Group Well-Being: Morale from a Positive Psychology Perspective. Applied Psychology. 2008;57(s1):19-36.

49. Britt TW, Dickinson JM, Moore D, Castro CA, Adler AB. Correlates and consequences of morale versus depression under stressful conditions. Journal of Occupational Health Psychology. 2007;12(1):34-47.

50. Evans L. Delving Deeper into Morale, Job Satisfaction and Motivation among Education Professionals: Re-examining the Leadership Dimension. Educational Management & Administration. 2001;29(3):291-306.

51. Hart PM. Teacher quality of work life: Integrating work experiences, psychological distress and morale. Journal of Occupational and Organizational Psychology. 1994;67(2):109-32.

52. Gulliver P, Towell D, Peck E. Staff morale in the merger of mental health and social care organizations in England. J Psychiatr Ment Health Nurs. 2003; 10(1); 101-7.

53. Johnson S, Wood S, Paul M, Osborn D, Wearn E, Lloyd-Evans B, et al. Inpatient Mental Health Staff Morale: a National Investigation. 2011. NIHR Service Delivery and Organisation Programme.

54. Totman J, Hundt G, Wearn E, Paul M, Johnson S. Factors affecting staff morale on inpatient mental health wards in England: A qualitative investigation. BMC Psychiatry.2011;11:68.

55. Johnson S, Osborn D, Araya R, Wearn E, Paul M, Stafford M, et al. Morale in the English mental health workforce: Questionnaire survey. Br J Psychiatr. 2012;201(3); 239-46.

56. Mistry H, Levack WM, Johnson S. Enabling people, not completing tasks: patient perspectives on relationships and staff morale in mental health wards in England. BMC Psychiatry. 2015;15:307.

57. Napier J, Clinch M. Job strain and retirement decisions in UK general practice. Occupational Medicine. 2019;69(5): 336-41.

58. Wang JY, Zhuang HL, Chiou JY, Wang CW, Wang CY, Liu LF. Exploring factors influencing the work-related morale for certified nursing assistants in hospice care: A structural equation modeling study. PloS one. 2018;13(10):e0206281.

59. Carmel S, Ramsey S, Nguyen VM, Haddaway NR, Gutowsky LF, Wilson AD, et al. The will-to-live scale: development, validation, and significance for elderly people. Aging Ment Health. 2017;21:289-96.

60. McVicar A. Scoping the common antecedents of job stress and job satisfaction for nurses (2000–2013) using the job demands–resources model of stress. Journal of Nursing Management. 2016;24(2):E112-E36.

61. Turnell A, Rasmussen V, Butow P, Juraskova I, Kirsten L, Wiener L, et al. An exploration of the prevalence and predictors of work-related well-being among psychosocial oncology professionals: An application of the job demands-resources model. Palliat Support Care. 2016;14(1):33-41.

62. Spence Laschinger HK, Grau AL, Finegan J, Wilk P. Predictors of new graduate nurses' workplace well-being: testing the job demands-resources model. Health Care Manage Rev. 2012;37(2):175-86.

63. Jourdain G, Chenevert D. Job demands-resources, burnout and intention to leave the nursing profession: a questionnaire survey. Int J Nurs Stud. 2009:47: 709-22.

64. Heuser CC, Gibbins KJ, Herrera CA, Theilen LH, Holmgren CM. Moms in medicine: Job satisfaction among physician-mothers in obstetrics and gynecology. Work. 2018;60:201-7.

65. Spiegel DA, Smolen RC, Jonas CK. An examination of the relationships among interpersonal stress, morale and academic performance in male and female medical students. Soc Sci Med. 1986;23(11):1157-61.

66. Anzai E, Douglas C, Bonner A. Nursing practice environment, quality of care, and morale of hospital nurses in Japan. Nursing & health sciences. 2014;16(2):171-8.

67. Cox KB. The effects of unit morale and interpersonal relations on conflict in the nursing unit. Journal of advanced nursing. 2001;35(1):17-25.

68. Fletcher E, Abel GA, Anderson R, Richards SH, Salisbury C, Dean SG, et al. Quitting patient care and career break intentions among general practitioners in South West England: findings of a census survey of general practitioners. BMJ open. 2017:7(4):e015853.

69. Gilliland AE, Sinclair H, Cupples ME, McSweeney M, Mac Auley D, O'Dowd TC. Stress and morale in general practice: a comparison of two health care systems. Br J Gen Pract. 1998;48(435):1663-7.

70. Nocon RS, Fairchild PC, Gao Y, Gunter KE, Lee SM, Quinn M, et al. Provider and Staff Morale, Job Satisfaction, and Burnout over a 4-Year Medical Home Intervention. J Gen Intern Med. 2019;34(6):952-9.

71. McKinstry B, Porter M, Wrate R, Elton R, Shaw J. The MAGPI (Morale Assessment in General Practice Index): A new way for doctors to self-assess their morale. Education for Primary Care. 2004;15:231-41.

72. Chandra S, M Wright S, Ghazarian S, M Kargul G, E Howell E. Introducing the Hospitalist Morale Index: A New Tool That May Be Relevant for Improving Provider Retention.J Hosp Med. 2016;11(6):425-31.

73. MacRobert M, Schmele JA, Henson R. An analysis of job morale factors of community health nurses who report a low turnover rate. The research. J Nurs Adm. 1993;23(6):22-7.

74. Yang KP, Huang CK. The effects of staff nurses' morale on patient satisfaction. J Nurs Res. 2005;13(2):141-52.

75. Grieve S. Measuring morale - Does practice area deprivation affect doctors' well-being? Br J Gen Pract. 1997; 47(422): 547-52.

76. Galeazzi GM, Delmonte S, Fakhoury W, Priebe S. Morale of mental health professionals in Community Mental Health Services of a Northern Italian Province. Epidemiol Psichiatr Soc. 2004;13(3):191-7.

77. Priebe S, Fakhoury WK, Hoffmann K, Powell RA. Morale and job perception of community mental health professionals in Berlin and London. Social psychiatry and psychiatric epidemiology. 2005;40(3):223-32.

78. Reininghaus U, Priebe S. Assessing morale in community mental health professionals: a pooled analysis of data from four European countries. Social psychiatry and psychiatric epidemiology. 2007;42(3):237-43.

79. Bowers L, Allan T, Simpson A, Jones J, Whittington R. Morale is high in acute inpatient psychiatry. Social psychiatry and psychiatric epidemiology. 2009;44(1):39-46.

80. Caravella RA, Robinson LA, Wilets I, Weinberg M, Cabaniss DL, Cutler JL, et al. A Qualitative Study of Factors Affecting Morale in Psychiatry Residency Training. Acad Psychiatry. 2016;40: 776-82.

81. Chen JY. Morale and role strain of undergraduate nursing students in a pediatric clinical setting. J Nurs Res;18:144-53.

82. Spencer K, Foster PER, Whittamore KH, Goldberg SE, Harwood RH. Staff confidence, morale and attitudes in a specialist unit for general hospital patients with dementia and delirium—a qualitative study. International Journal of Geriatric Psychiatry. 2014;29(12):1315-7.

83. Huby G, Gerry M, McKinstry B, Porter M, Shaw J, Wrate R. Morale among general practitioners: qualitative study exploring relations between partnership arrangements, personal style, and workload. BMJ. 2002;325(7356):140.

84. Hartley S, Macfarlane F, Gantley M, Murray E. Influence on general practitioners of teaching undergraduates: qualitative study of L ondon general practitioner teachers. BMJ. 1999;319(7218):1168-71.

85. Rucker L, Shapiro J, Fornwalt C, Hundal K, Reddy S, Singson Z, et al. Using focus groups to understand causes for morale decline after introducing change in an IM residency program. BMC Med Educ. 2014;14:132.

86. Singh R, Kirtley J, Lakhani D, Carr S. Listening to junior doctors: exploring morale in a large NHS trust. Future Healthc J. 2019;6.:179.

87. Dowling S, Last J, Finnegan H, Daly P, Bourke J, Hanrahan C, et al. Impact of participation in continuing medical education small group learning (CME-SGL) on the stress, morale, and professional isolation of rurally-based GPs: a qualitative study in Ireland. BJGP Open. 2019;3(4):bjgpopen19X101673.

88. McKinstry B, Walker J, Porter M, Fulton C, Tait A, Hanley J, et al. The impact of general practitioner morale on patient satisfaction with care: a cross-sectional study. BMC Fam Pract. 2007;8:57.

89. Montoro-Rodriguez J, Small JA. The role of conflict resolution styles on nursing staff morale, burnout, and job satisfaction in long-term care. J Aging Health. 2006.;18:385-406.

90. McFadzean F, McFadzean E. Riding the emotional roller-coaster: a framework for improving nursing morale. J Health Organ Manag. 2005;19(4-5):318-39.

91. Day GE, Minichiello V, Madison J. Nursing morale: what does the literature reveal? Australian health review : a publication of the Australian Hospital Association. 2006;30(4):516-24.

92. Day G, Minichiello V, Madison J. Nursing morale: Predictive variables among a sample of Registered Nurses in Australia. J Nurs Manage. 2007;15(3):274-84.

93. Callaghan M. Nursing morale: What is it like and why? Journal of advanced nursing. 2003;42(1):82-9.

94. Richards DA, Bee P, Barkham M, Gilbody SM, Cahill J, Glanville J. The prevalence of nursing staff stress on adult acute psychiatric in-patient wards. A systematic review. Social psychiatry and psychiatric epidemiology. 2006;41(1):34-43.

95. Cahill J, Gilbody S, Barkham M, Bee P, Richards D. Systematic review of staff morale in inpatient units in mental health settings. 2004. National Co-Ordinating Centre for NHS Service Delivery and Organisation R&D, London, UK.

96. Gilbody S, Cahill J, Barkham M, Richards D, Bee P, Glanville J. Can We Improve the Morale of Staff Working in Psychiatric Units? Journal of Mental Health. 2009;15:7-17.
